# Failure mechanism of TRSS mode in landslides induced by earthquake

**DOI:** 10.1038/s41598-020-78503-y

**Published:** 2020-12-07

**Authors:** Renli Liu, Yanhui Han, Jun Xiao, Tao Wang

**Affiliations:** 1 State Key Laboratory of Water Resources and Hydropower Engineering Science, Wuhan University, Wuhan, 430072 China; 2 Department of Civil, Environmental and Geo-Engineering, University of Minnesota, Minnesota, MN 55455 USA

**Keywords:** Natural hazards, Seismology, Geophysics, Civil engineering

## Abstract

Investigation of the slopes in the Wenchuan earthquake shows that tension failures appear in the upper part in many landslides. The typical failure mode can be generalized as “tensile-rupture and sheared-sliding” (TRSS). In this paper, the distinct element method (DEM) is employed to simulate the gradual failure process of the Tangjiashan landslide under the excitation of the Wenchuan earthquake. It is found that the first failure is the appearance of deep tension cracks on the top, and then shearing slip along the bottom. The posterior fracture is deep, steep, and rough, and the bottom shear slip surface has a relatively gently dip. The simulation shows the failure mode of TRSS in the slope can be well reproduced, and the geological and mechanical mechanisms can be revealed in the DEM model.

## Introduction

The term “landslide” describes a wide variety of processes that result in the downward and outward movement of slopes consisting of rock, soil, tailings, or a combination of them. Seismically induced landslide in mountainous regions is one of the most serious geological hazards. Slope failures resulting from earthquakes often cause extensive damage to lifeline systems and transportation networks^1^. A large earthquake is capable of triggering thousands of landslides over a large area^2,3^. In general, the presence of discontinuities has a profound influence on the stability of rock slopes^4,5^. A novel method for correcting scanline-observational bias of discontinuity orientation is proposed to for discontinuities modelling^6^.

For the problems they care about, many researchers have put forward corresponding classification methods. Some of them are listed in Table 1. Discussion of the method of classification is not within the scope of this article. Rather we shall focus on the failure mechanism behind a common failure mode in the Wenchuan earthquake, which can be used to get a more reasonable classification. The one developed by Varnes^7^ has been most commonly adopted, in which a landslide is described by the type of movement and materials. Keefer^2^ categorized earthquake-induced landslides into two types, landslides in rock and landslides in soil. Landslides in rock is further divided into two classes, i.e., (a) disrupted slides and falls; (b) coherent slides. Hutchinson^8^ provided a classification considering geology and hydrogeology environment. A European classification was developed from the EPOCH project, which was suitable for European conditions^9^. Dikau et al.^10^ provided a classification of the landslide, combining the opinions of Hutchinson^8^ and EPOCH^9^.

**Table 1:** Summary of landslide classification systems.

Provider	Application	Material	Movement	Instability Mechanism	Comments
Coates and Gyenge^15^	General			○	Classification of slope failures by mechanisms
Varnes^7^	General	○	○	○	The most commonly adopted systems
Keefers^2^	Earthquake	○	○		Based on the principles and terminology of Varnes
Hutchinson^8^	General	○	○	○	Geology and hydrogeology environments are considered
EPOCH^9^	General	○	○		Simple and suitable for European condition
Dikau^10^	General	○	○		Compatible with Hutchinson (1988) and EPOCH (1993)
Khazai and Sitar^16^	Earthquake (Chi-Chi)	○	○		The landslides triggered by the Chi-Chi earthquake are classified within four broad categories
Huang^13^	Earthquake (Wenchuan)		○	○	The main types were divided in terms of the failure mechanism and dynamic features
Dai et al.^14^	Earthquake (Wenchuan)		○	○	Four broad categories considering depth of landslides and failure modes
Xu et al.^17^	Earthquake (Wenchuan)		○	○	Genetic modes of landslides were established through surveying the deformation features

Items from column 3 to 5 are classification basis, the presence is symbolized by a circle.

Located between pacific seismic zone and Euro-Asia seismic zone, China suffers from frequent earthquakes and endure heavy disasters. According to the characteristics of slope movement, the landslides in the southeast of China can be classified into four types: pushing landslide, hauling landslide, bedding landslide and avalanching landslide^11^. Based on the geologic condition, the slope failure modes can also be described by whether it is obviously controlled by discontinuities^12^. In the occurrence of the Wenchuan earthquake in 2008, many catastrophic landslides were triggered in the earthquake zone. Huang et al*.*^13^ defined a classification of slope failure under the Wenchuan earthquake considering the failure mechanism and dynamic process. This approach is more targeted but may encounter difficulties in the application and promotion. However, considering geo-mechanics and dynamic features, Dai et al.^14^ classified the landslides in the Wenchuan earthquake into four broad categories: shallow, disrupted landslides; rock falls; deep-seated landslides; and rock avalanches.

Xu et al.^17,18^ developed a method to classify the landslides induced by the Wenchuan earthquake into five types; it is changed to four types later as adjustment. In their system, the basic and internal deformation and failure mode in the large-scale landslides induced by strong shock are classified based on the type of tension-cracks and shearing sliding. In their investigations, Zheng et al.^19^, Xu et al.^17^and Yan et al.^20^ found that in the Wenchuan earthquake the tensional failures appear in the upper part of the most landslides, and in some slopes some rock and soil masses were even thrown out through the top fractures. The impact of the earthquakes on the slope can be assessed quantitatively, but is still very difficult to achieve, considering the complexity and variety of landslide types^21^.

Ten years after Wenchuan earthquake, we can have new insights into the failure mechanism of the landslides induced by the earthquake. In this study, we use term “tensile-rupture and sheared-sliding” (TRSS) to describe this kind of destruction phenomenon, that is, under the earthquake condition, the first location of the slope failure is the appearance of tension cracks on the crest, and then shearing sliding along the bottom slip surface; after the tensile fracture and shear fissure are connected, landslide will be formed. The failure mechanism of landslides induced by the earthquake has been mostly qualitatively analyzed by the geologic field exploration. However, there is little or no information available in the literature on synthetic engineering geologic analysis and dynamic response computation of landslides under excitation of an earthquake. Based on the geologic data and analysis methods proposed by the early researchers and the experience in numerical modeling in recent years, we applied Distinct Element Method^22^ to model the progressive failure process of the rock slope loaded by a strong seismic shock and analyze the TRSS failure mechanism of the landslide during earthquake.

## Geomechanics exploration of TRSS

Analytical solutions for the elastic medium under gravity with an irregular surface of a slope are difficult. The problem may be studied by the photoelastic method or finite element methods^23^. Under gravity loading, the stresses of the shallow part of the slope are: the vector of maximum principal stress is basically parallel to the slope surface and the vector of minimum principal stress is basically perpendicular to the slope surface. In this stress state, shear stress concentration occurs near the slope toe, as confirmed by some existing studies, e.g., in Jumikis^24^. It is also possible that there will be shearing deformation and failure along the middle, gentle dip discontinuities of the slope.

Zheng et al.^19^ applied FLAC^3^^D^ strength reduction with the functions of tensile and shear failure to study the failure of a slope under earthquake loading. The study shows that the dynamic failure of the slope is a composition of the tensile failure in the upper part and the shear failure in the lower part instead of shear sliding failure. Xu et al.^17^ found from the vibration platform experiments that the strong inertial force produced from the earthquake is the main cause of landslides. The strong horizontal seismic inertia force induces the vertical fracture perpendicular to the direction of the seismic force on the slope top firstly, at the same time, it also drives the fracture of the lateral deformation along the horizontal direction. The nearly horizontal outward tension-shearing fracture is generated, and the global instability of the slope occurs eventually.

Li and Rice^25^ think that the most important changes in their perception and understanding of rock fracture in the earth's crust have come about because of the application of fracture mechanics in geophysics. The concepts are equally valid and applicable in many other fields of research, such as in geotechnical engineering where the failure of jointed rock masses and failure in soil slopes are of major concern^26^. In essence, fracture mechanics concerns the study of stress concentrations caused by sharp tipped flaws and the conditions for the propagation of these flaws. The theory of linear elastic fracture mechanics (LEFM) uses an analytical approach that will provide useful analytical steps. This method is valid provided that any region of non-linear behavior is negligibly small compared with the length of the crack and the dimensions of the cracked body^27^. There are three basic modes of crack tip displacement, which are termed model-I, tensile, mode-II, in-plane shear and mode-III, anti-plane shear. A key concept in the use of fracture mechanics is that extension of a fracture will occur once a critical value of stress intensity factor, *K*_*c*_, or crack extension force, *G*_*c*_, has been reached or exceeded. When θ = 0, the *K*_*c*_ value for mode-I and Mode-II can be defined as

1 $$ K_{I} = \mathop {\lim }\limits_{r \to 0} [\sigma_{y} (2\pi r)^{{{1 \mathord{\left/ {\vphantom {1 2}} \right. \kern-\nulldelimiterspace} 2}}} ] $$

2 $$ K_{II} = \mathop {\lim }\limits_{r \to 0} [\tau_{xy} (2\pi r)^{{{1 \mathord{\left/ {\vphantom {1 2}} \right. \kern-\nulldelimiterspace} 2}}} ] $$

where *σ*_*y*_ is the tensile stress normal to the crack surface, *r* is the distance measured from the crack.

For fracture in each of the fundamental modes of crack tip displacement, the crack extension force for plane strain and assuming linear elasticity is given by

3 $$ G_{I} = K_{I}^{2} (1 - p^{2} )/E $$

4 $$ G_{II} = K_{II}^{2} (1 - p^{2} )/E $$

where *p* is Poisson’s ratio, *E* is Young’s modulus.

Once the critical value of *K* or *G* has been reached, the crack propagation is known as fast or catastrophic fracture. There are several kinetic laws for crack extension and they share the common form

5 $$ v = v(K,G) $$

where *v* is the crack velocity. The specific form of the crack velocity dependence on *K* or *G* depends on the precise mechanism whereby the energy barrier to crack extension is overcome. The subcritical crack growth is a general view of crack growth during long-term loading. The cracks formed under earthquake should belong to catastrophic crack propagation. With the help of theory of LEFM, the velocity of crack expansion under earthquake can only be calculated primarily, it’s limited to the several simple conditions. It seems that numerical simulations will result in more comprehensive results.

**Figure 1:**
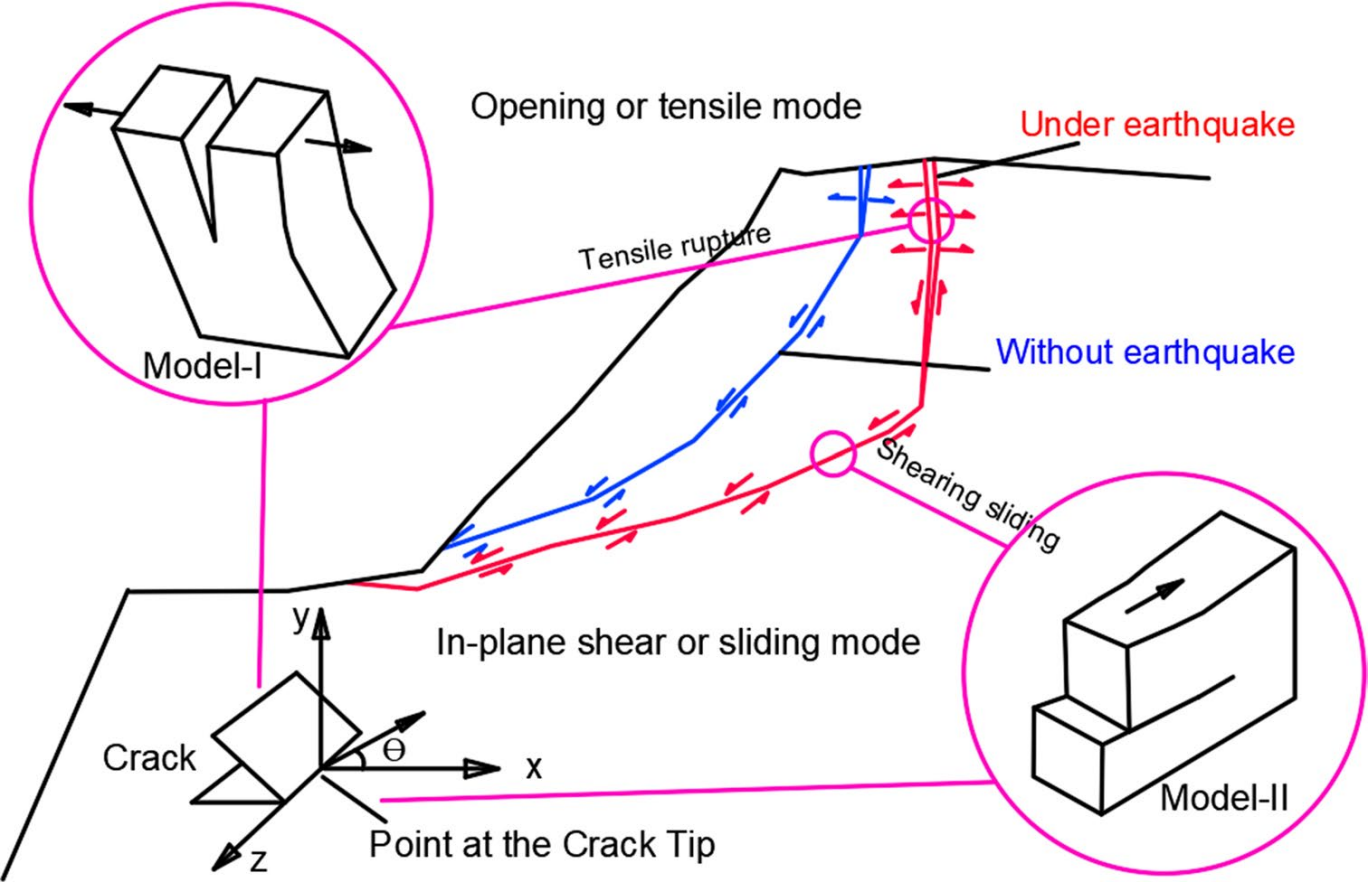
Schematic of the different sliding surfaces under static and earthquake loading conditions. Tensile rupture fractures are observed at the top of the slope under earthquake loading condition and sheared sliding is formed after tensile rupture. The stress loading modes and crack coordinates system are also demonstrated.

It can be inferred that, under the condition of strong earthquake and the amplification effect of elevation, the stress produced by horizontal seismic wave will be much higher than the tensile strength of rock mass, therefore, the vertical fracture surface nearly parallel to the slope surface is easy to be generated (Fig. 1). Evidently, under seismic conditions, stress state and response of rock mass differs significantly in many aspects from conventional static gravitational loading condition. Under the gravitational loading condition, the failure mode of the slope is shearing sliding along the weak surface, and then the tensile cracks of small-scale occur at the trailing edge. the shear slip surface grows and demonstrates nearly mode of pure shear; the rear fracture is shallower, and the scale is relatively smaller, showing the deformation and failure characteristics of the bottom shear slip. In contrast, under the earthquake conditions, the first failure is the appearance of deep tension cracks (Mode-I) on the top, and then shearing slip along the bottom (Mode-II). The rear fracture is deep, steep and rough, and the bottom shear slip surface has a relatively gently dip, and it is characterized by shearing-tension, and showing tensile failure of the edge.

**Figure 2:**
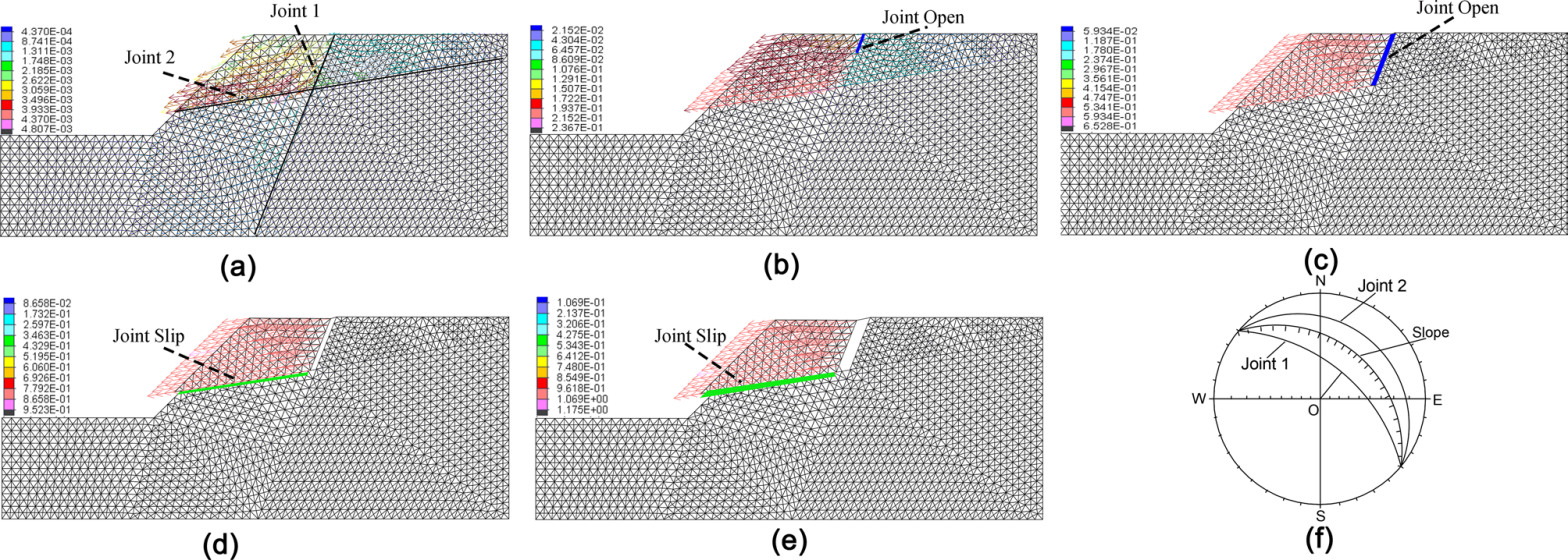
Five stages (**a**–**e**) of the development process of landslide simulated by UDEC. The colored vector indicates the displacement change (unit: m). The TRSS mode is formed in an ideal jointed rock slope. (**f**) Stereographic projection of the jointed slope system. We produced the figures using UDEC (Ver.6.0) software (https://www.itascacg.com/software/UDEC).

Hoek and Bray^28^ provided that the presence of tension fractures on the top of a slope should be taken as an attention of potential failure. This conclusion is also applicable to the analysis of slope stability under seismic conditions. It is possible to use UDEC interfaces to model Mode I crack propagation^29^. We have used UDEC^30^ to analyze the occurrence and development of the tensile-shear failure mode in seismic dynamic loading of an ideal jointed rock slope(Fig. 2). After applying seismic waves, the bottom sliding surface runs through with the combination of Joint 1 and Joint 2. It can be seen from this case that the failure of the slope is developing mainly along the joint surface. The cracks occur at the slope crest at first and then open along Joint 1. Until the open joint is contacted with Joint 2, Joint 2 begins to slip. Finally, as Joint 1 and Joint 2 are intersected with each other, the slope fails at the TRSS mode (Fig. 2e). It can be seen that mechanical behavior is joint open in Joint 1 (Mode-I) and joint slip in the Joint 2 (Mode-II). We can draw the same conclusion using a stereographic projection method (Fig. 2f). These simulation results are consistent with the experimental results^31^. There are 3 great circles in Fig. 2f, which represent the slope surface, joint surface 1 and joint surface 2. When the slope surface is located between the joint surface 1 and the joint surface 2, the slope is in a state of sliding failure^28^. It is found that the discontinuities distribution and strong seismic force are important reasons for the formation of TRSS. It can be speculated that the strong seismic force will make the slope produce deep tension fractures, which will connect with the around discontinuities and shorten the length of the anti-sliding part.

## Failure research of the Tangjiashan landslide

### Geomorphologic and geological setting

**Figure 3:**
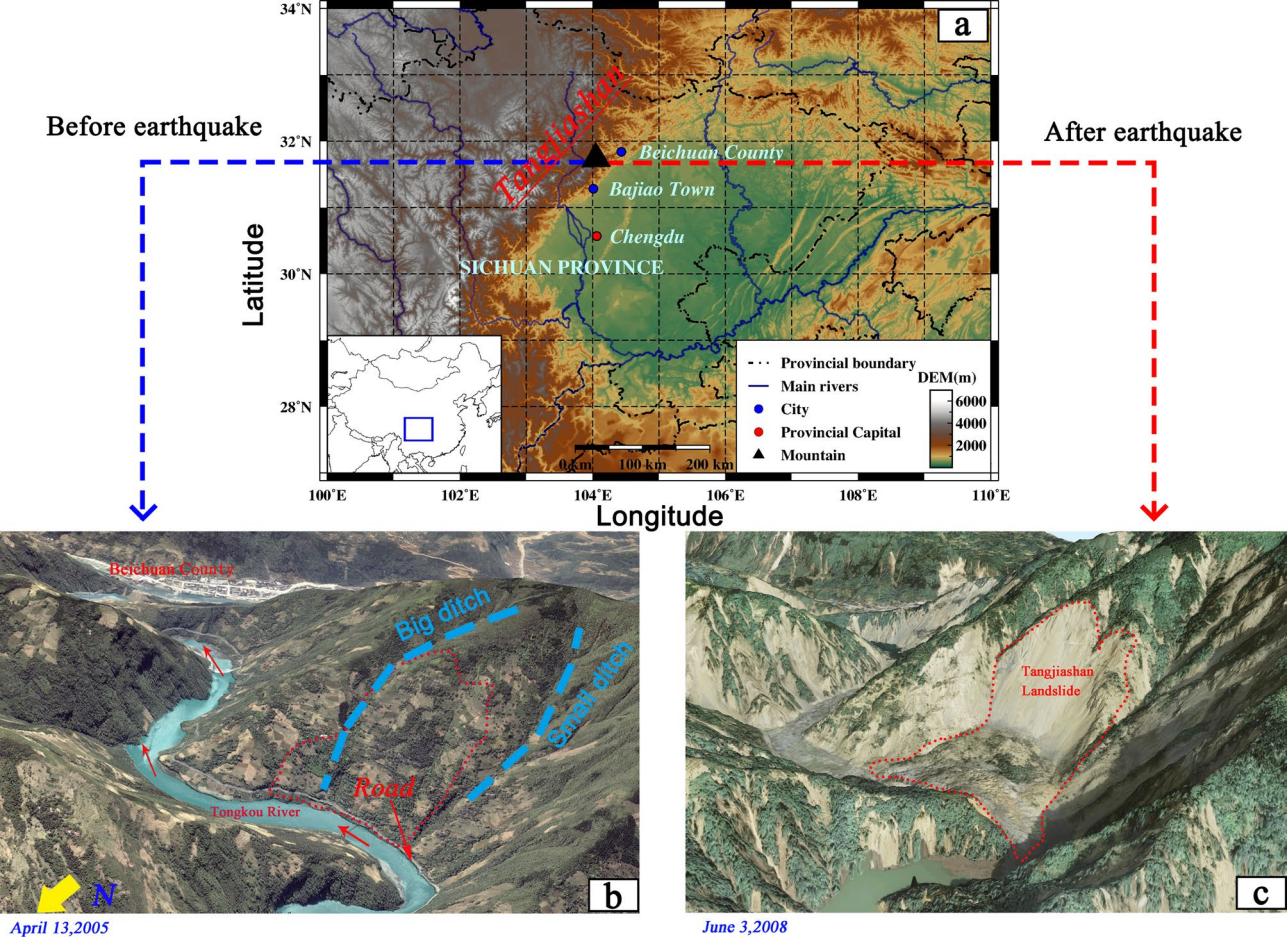
The digital elevation model and remote sensing model of the research zone: (**a**) location of the Tangjiashan landslide; (**b**) before the earthquake (from upstream); (**c**) after the earthquake (from upstream). The gmt-5.4.5 (https://github.com/GenericMappingTools/gmt/releases/tag/5.4.5) was used to generate the (**a**), and Google Earth Pro 7.3 (https://www.google.com/intl/en/earth/) was used to generate the (**b**,**c**).

**Figure 4:**
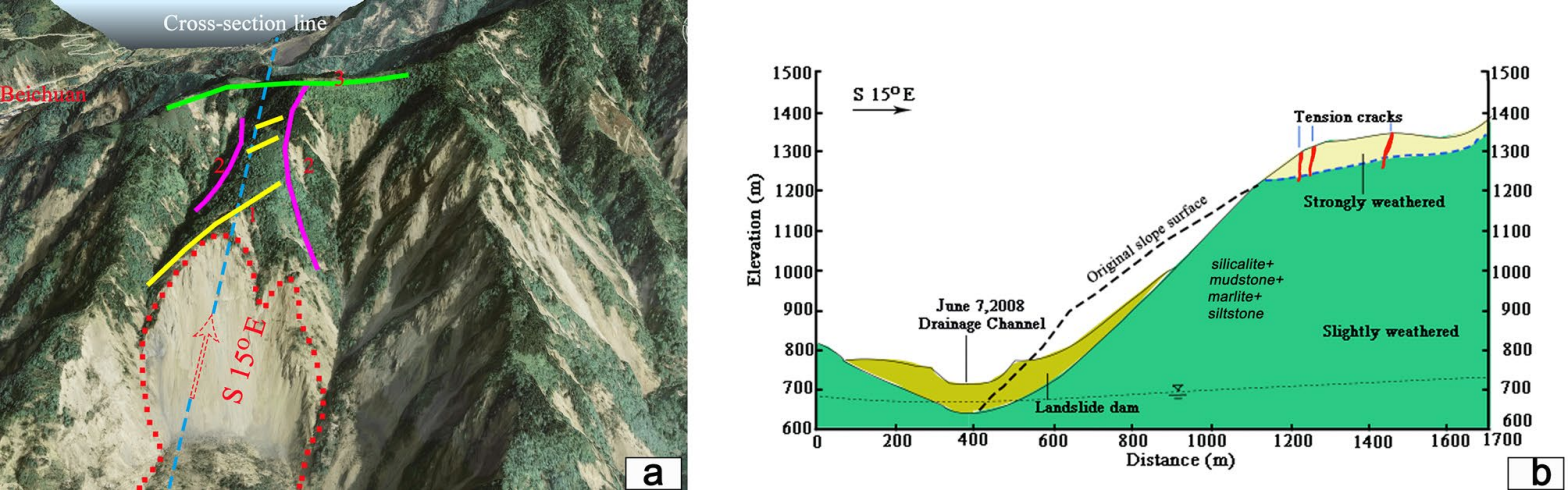
(**a**) Distribution of cracks in the residual mountain after landslide; (**b**) Engineering geological profile through the Tangjiashan landslide and landslide dam. Google Earth Pro 7.3 (https://www.google.com/intl/en/earth/) was used to generate the (**a**).

The Tangjiashan landslide is located on the right bank of Tongkou River in Sichuan Province, China (Fig. 3a), around 125 km from 2008 Wenchuan earthquake epicenter of Yingxiu Town^32^. The landslide is about 2.5 km away from Yingxiu—Beichuan part of Longmenshan Central Fault Zone (LCFZ)^33^. The Tongkou River snakes through the landslide area (Fig. 3b). The water level in the Tongkou River during summer was about 664 m; the river surface was 100 m to 130 m in width, and 0.5 m to 4 m in depth. Before the earthquake, the terrain gradient of the slope was about 40°, the elevation of the slope toe was about 665 m and the elevation the slope crest was about 1400 m above sea level (Fig. 4b).

The 2008 Wenchuan earthquake (*M*_w_ 8.0) occurred along the LCFZ with an epicenter depth of 19 km (shallow earthquake). The seismic intensity in the area of the landslide was XI. Tangjiashan slide was induced by the earthquake almost simultaneously, and it was the most hazardous of the 40 or so valley-blocking landslides triggered by the shock of the world^34^. The main strata of the slope are the lower Cambrian system/(∈_1_), contains siltstone, silicalite, marlite, and mudstone. The rocks are well bedded striking EW and dip moderately to steeply towards the north. The landslide also involved the alluvial deposit of Quaternary sediments (Q^al^). These strata of the slope are dipping outward (North direction) and forming a bedding slope of steep incline (Fig. 4b). In contrast, the other side of the valley forms a reverse dip slope.

The true-color image in Fig. 3c shows the landslide dam-created lake on June 3, 2008. Compared to the image taken on April 13, 2005 as shown in Fig. 3b, the Tongkou River has been blocked up. The road (the white line in Fig. 3b) that once ran alongside the river has been buried after slope failure.

Combining photograph interpretation by helicopter low altitude flight with the field geological surveying nearby the remnant mountain body behind the scarp after the landslide, it indicates that there are mainly three sets of cracks distributing on the surface of the remnant mountain body after earthquake, which are all tension cracks with the opening width of about 10 ~ 50 cm. The distribution of three sets of fractures is shown in Fig. 4a: (1) the set along the strike direction of N40°–50°E or N70°E-EW with the extended length from 20 to 200 m (dip angle is between 60° and 80°), paralleled to the trailing edge of landslide, showing unloading tension failure towards the free face of Tongkou river (The yellow line in Fig. 4a); (2) the set along the strike direction of N10° W–N10° E, paralleled to the trend of the Big Ditch and the Small Ditch valleys, belonging to unloading tension failure towards the free faces of the Big Ditch and the Small Ditch (The purple line in Fig. 4a); (3) the set along the strike direction of N70°W, distributing on the ridge lines, paralleled to the trend of the ridge lines, belonging to the seismic crack in ridge lines of the steep slopes (The green line in Fig. 4a). It can be diagnosed that there existed tension cracks indication of the slope crest before earthquake occurrence, and the seismic loading leads to the development and initiation of tension cracks. Tension cracks expand downward to cause the development of shear cracks and all tracks ran throughout at last. The Tangjiashan landslide is a typical failure mode of TRSS from the field investigation.

After the landslide occurrence, with a volume of 20 million m^3^, the debris of landslide mass accumulated and formed the landslide dam with a thickness of 80 to 125 m. The landslide that dammed the river covers about 800 m along the river bend and about 650 m along transverse river direction. About 100 people lost their lives in this disaster^18,35^. It is supposed to be residents, vehicles, and pedestrians on the roads. If the dam breaks down, the water (240 million m^3^) will barrel down into the river. The people in Beichuan County (around 6.5 km downstream) will suffer the worst battering if the blockage cannot be excavated timely. The engineer used digging equipment and explosives to dig a drainage channel in the dam to ease water from the dangerous lake and relieve the pressure behind it. On June 7, 2008, the channel was completed and the water can flow downstream.

### Seismic input and material properties

UDEC is used as a computing tool here. As for the Tangjiashan slope model, the blocks in UDEC are assumed as the elastic-perfectly plastic bodies following the Mohr–Coulomb failure criterion this time; the joints are considered as face-contacted Coulomb sliding model. In the DEM, a rock mass is represented as an assembly of discrete blocks. Joints are viewed as interfaces between distinct bodies. The contact forces and displacements at the interfaces of a stressed assembly of blocks are found through a series of calculations that trace the movements of the blocks. In turn, movements result from the propagation through the block system of disturbances caused by applied loads or body forces. This is a dynamic process in which the speed of propagation depends on the physical properties of the model^36^. The mechanical parameters of rock masses and joints are listed in Tables 2 and Table 3, respectively. The mechanical parameters are obtained through the results of laboratory physical mechanics experiments, with the consideration of geological characteristics of the slope.

**Table 2:** Rock masses mechanical parameters of the slope.

Rock type	Density (Kg/m^3^)	Bulk modulus (GPa)	Shear modulus (GPa)	Friction angle (°)	Cohesion (MPa)
Strongly weathered	2650	5.56	4.17	32	0.3
Slightly weathered	2750	6.67	5.0	35	0.4

**Table 3:** Discontinuities mechanical parameters.

Category	Normal stiffness (GPa/m)	Shear stiffness (GPa/m)	Tensile strength (MPa)	Friction angle (°)	Cohesion (MPa)
Bedding plane	2.32	1.73	0.05	30	0.1
Joint	1.81	0.92	0.03	25	0.08

Bajiao town seismic station is the nearest station recording the seismic wave to the landslide (Fig. 3a), the distance is about 75 km. So, field measures acceleration data of the station is used as an input seismic wave. The seismic wave of 120 s is chosen from the seismic wave (The strong vibrations hold about 40 s), and the acceleration amplitude is 6.33 m/s^2^ in the vertical direction and 5.81 m/s^2^ in the horizontal direction. Cui et al.^32^ and Cao et al.^33^ did not explain the specific channel of horizontal acceleration wave. Xu et al.^37^ pointed out the horizontal acceleration data he adopted was in an east–west direction. He considered the two horizontal accelerations were equal and the vertical acceleration was 2/3 of the other directions. In this study, we used three accelerations collected form Bajiao town seismic station. The two horizontal accelerations are synthesized following the principle of vector summation as the input horizontal wave. The input vertical acceleration is real data from Bajiao town seismic station. The dynamic analysis conditions are listed in Table 4.

**Table 4:** Dynamic analysis conditions for Tangjiashan landslide.

Boundary conditions			Dynamic load		
Bottom	Left and right	Upper	Horizontal	Vertical	Damping type
Quiet boundary	Free field boundary	Unconstrained	WE + NS components orthogonal transformed	UD component	Local/0.08

For deformable blocks, the equations of motion are expressed by the following equation, which incorporates local damping:

6 $$ \dot{u}_{i}^{{\left( {t + \Delta t/2} \right)}} = \dot{u}_{i}^{{\left( {t - \Delta t/2} \right)}} + \left( {F_{d} } \right)_{i} \frac{\Delta t}{{m_{n} }} $$

where

7 $$ \left( {F_{d} } \right)_{i} = \sum {F_{i}^{(t)} } - \alpha \left| \sum {F_{i}^{(t)} } \right|{\text{sgn}} \left( {\dot{u}_{i}^{t - \Delta t/2} } \right) $$

$F_{d}$ is the damping force; *α*_*d*_ is the local damping ratio; $F_{i}^{(t)}$ is the unbalanced force; $\dot{u}_{i}^{(t)}$ is the velocity at the grid point; $m_{n}$ is a fictitious nodal mass; and *∆t* is the time increment.

The solution scheme used for the distinct element method is conditionally stable. A limiting timestep that satisfies the stability criterion for both calculation of internal block deformation and inter-block relative displacement is determined. The timestep required for the stability of block deformation computations is estimated as

8 $$ \Delta t_{n} = 2\min \left( {\frac{{m_{i} }}{{k_{i} }}} \right)^{1/2} $$

where *m*_*i*_ is the mass associated with block node *i*; and *k*_*i*_ is the measure of the stiffness of the elements surrounding the node *i*.

**Figure 5:**
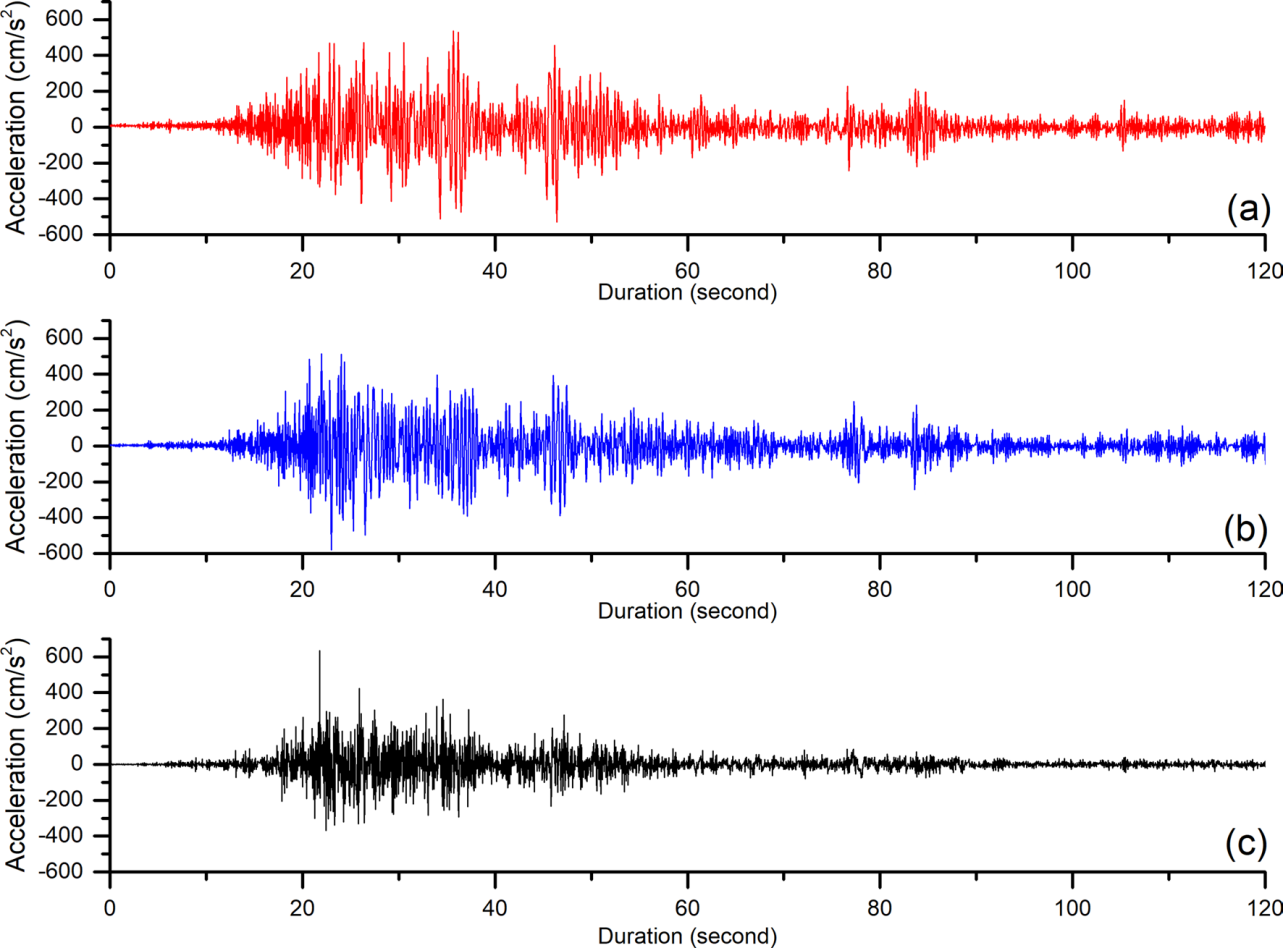
Acceleration-time curves of the Wenchuan seismic wave used in the model (Horizontal and vertical ground acceleration records of Bajiao town seismic station). (**a**) Horizontal: West–East component. Peak Ground Acceleration = 556.17 cm/s^2^ (**b**) Horizontal: North–South component. Peak Ground Acceleration = -581.59 cm/s^2^ (**c**) Vertical: UD component. Peak Ground Acceleration = 633.09 cm/s^2^.

The actual input seismic wave shown in Fig. 5 is applied in the horizontal and vertical direction of the model. Wave reflections at model boundaries are minimized by specifying quiet and free field boundary conditions.

### Analysis of the slope failure process and mechanism

**Figure 6:**
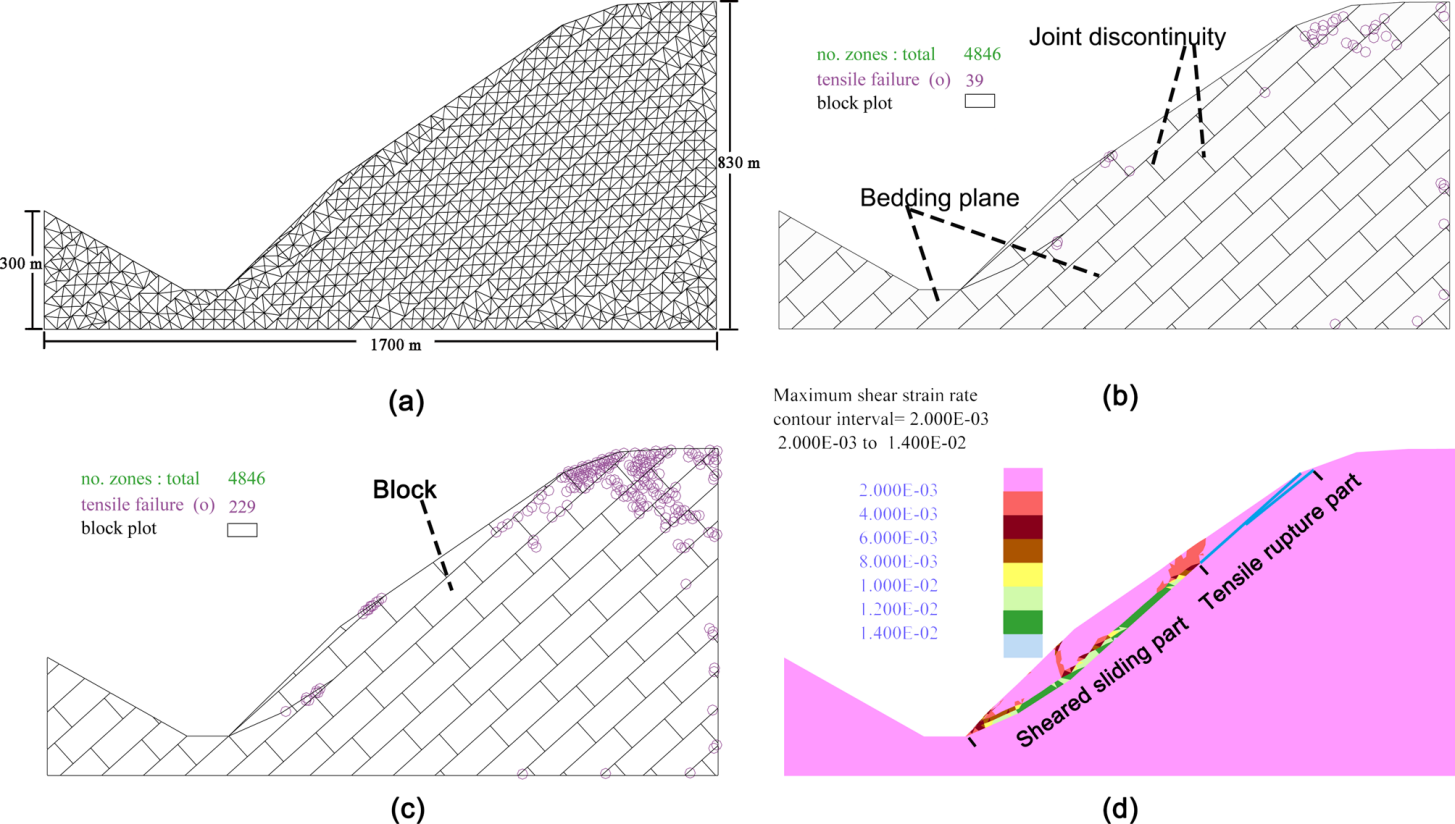
UDEC modeling of the Tangjiashan landslide. (**a**) Calculation zones of UDEC model, the blocks are discretized into deformable finite difference zones. The sizes of the model are also indicated. (**b**) Initial tensile failure state under gravity loading (purple circles indicate tensile failure zones). (**c**) Tensile failure state in the early stage under excitation of the Wenchuan seismic wave (purple circles indicate tensile failure). (**d**) Shear strain zones connected under excitation of the seismic wave, the tensile rupture part and sheared sliding part are marked along the sliding surface. We produced the figures using UDEC (Ver.6.0) software (https://www.itascacg.com/software/UDEC).

The UDEC model need to be at an initial force-equilibrium state under gravity loading before the seismic excitation is applied to the slope. Figure 6a shows the DEM model with calculation zones. UDEC can discretize blocks into triangular, constant-strain zones. The zones may follow an elastic or nonlinear constitutive law. Figure 6b shows the plastic failure zones of the Tangjiashan slope under the gravity loading. The tensile failure zones are mainly distributed on the slope crest. The seismic loading is applied after the initial stress equilibrium. Figure 6c shows the plastic failure zones in the early stage (20 s) under excitation of the Wenchuan seismic wave. The tensile failure zones increase obviously and the distribution on the slope crest is consistent with the tensile cracks found in the aerial photograph as shown in Fig. 4, where there were tensile cracks on the slope crest before the landslide took place. Figure 6d shows the tensile rupture part and the shear strain contour of slope in the early stage (20 s) under excitation of the Wenchuan seismic wave. It is observed that after the tensile rupture of the upper part of the slope, the shear strain zone run through the slope model. After the connection of the tensile rupture zone and sheared failure zone, the failure surface spread further from the slope crest to the slope toe basically along the contact zone between strong weathered and weak weathered rock strata. The increasing shear strain leads to the formation of slip surface and the slope toe locked segment is cut down and the through-failure surface is formed, and the whole mass above the sliding surface starts to move at a certain speed.

The slope goes into the sliding phase when the deformation accumulates to a certain degree. The high potential energy of the landslide body is converted into kinetic energy continuously during the sliding process, and the strong friction will produce huge heat. When the landslide body is sliding at a high velocity, the air under it is hard to evacuate rapidly and form the air cushion to sustain the landslide body. According to local villagers, the duration of airwave caused by the earthquake is less than 0.5 min^35^. The air cushion effect is similar to the Tsaoling landslide in Taiwan during the Chi-Chi earthquake^38^. Since the frictional resistance between the landslide body and the sliding surface is reduced by the action of air cushion, the landslide body tends to move at a high sliding velocity. The front of landslide body sharply slides down until it is blocked by the opposite slope which angel is 25°–40° and the high-speed landslide rapidly moves downwards and then climbs up the opposite slope. At the moment, the back of the landslide body is continuously sliding down until blocks the river and then, the dam-created lake is formed. That is, after the accumulation phase, a negative terrain between the toe and the head of the landslide is formed at the end.

**Figure 7:**
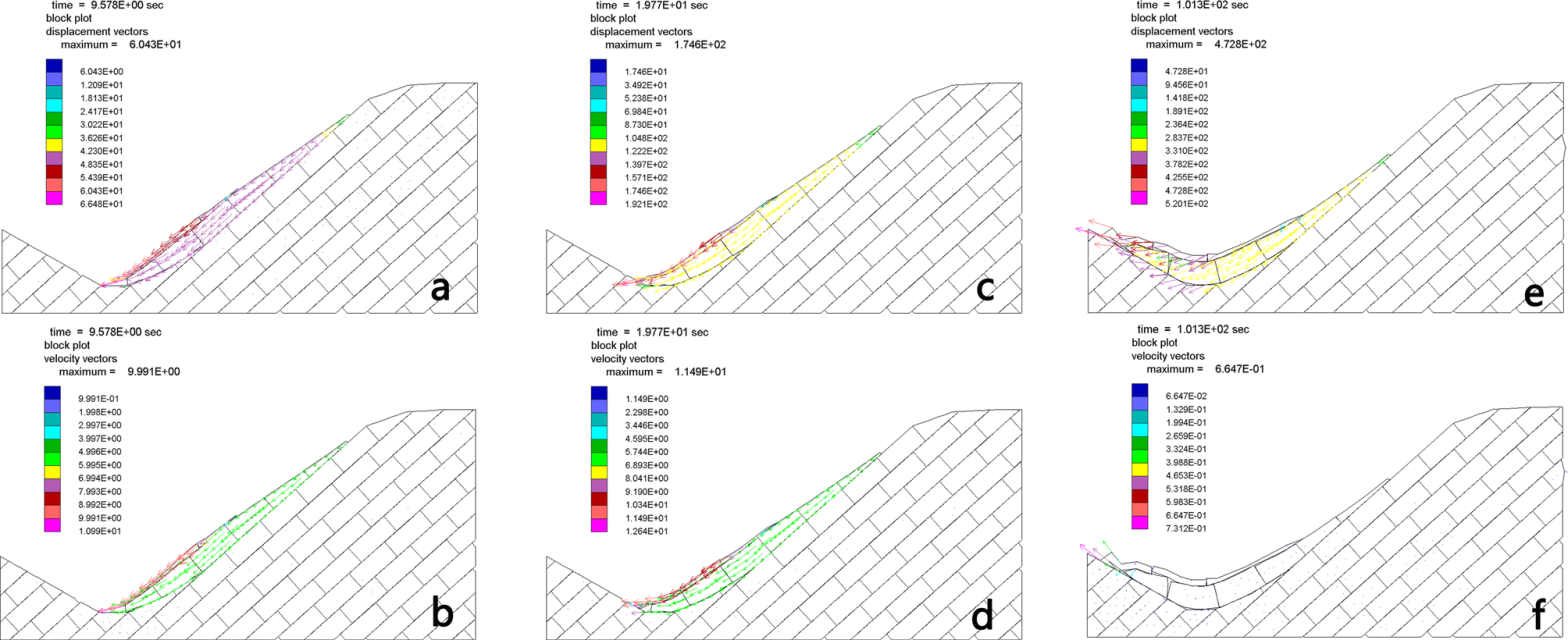
Block movement states of the Tangjiashan landslide at different times. After 100 s, landslide dam-created lake is formed. (**a**–**c**) are displacement vectors of different times; (**d**–**f**) are velocity vectors of different times. We produced the figures using UDEC (Ver.6.0) software (https://www.itascacg.com/software/UDEC).

The movement process of the landslide body during the whole sliding process can be observed clearly in the model as demonstrated in Fig. 7. At the dynamic simulation time of 10 s, the front of the landslide body starts to slide across a distance of 66 m (Fig. 7a,b). From 20 s, the whole sliding body reaches a certain speed (13 m/s) and forms sliding along the sliding surface (Fig. 7c,d). It is speculated that air cushion is mainly formed at this stage and this process lasted about 30 to 40 s. The high-speed landslide body receives the force from the air. In addition to the air resistance, the slide body also receives the action of the air lift, that is, a high-speed air cushion effect is generated between the slide body and the surrounding air. The high-speed landslide body receives the force from the air. In addition to the air resistance, the slide body also receives the action of the air lift, that is, a high-speed air cushion effect is generated between the slide body and the surrounding air. After 60 s, the dynamic accelerations become smaller obviously, the sliding body keeps moving downwards due to the inertial effect mainly. After 100 s, most part of sliding body tends to be stabilized, the blocks at the front of the sliding body continue to move with a small speed (0.7 m/s) (Fig. 7e,f). At around 120 s, the whole sliding body is nearly stabilized, and the permanent displacement is about 520 m. It needs to be noted that the landslide model in the UDEC calculation is simplified. It is a two-dimensional model, and we focused on the dominant discontinuity sets in the simulation. After the TRSS landslide mode is formed, the landslide body finally slides along the interface between the blocks. In the end, it seems to show bedding, which is related to the simplification of the UDEC model, but there is no doubt that TRSS is the core mechanism of landslide formation.

## Conclusions

The occurrence condition of TRSS is closely related to geological condition, earthquake wave and topographic condition, which can be investigated using the method that we developed in this study, although our focus here is to understand the failure mechanisms behind the observed failure mode. The pattern of TRSS is commonly observed in the Wenchuan earthquake area. It is reproducible in both the idealized conceptual model (Fig. 2) and the real model for the Tangjiashan landslide (Fig. 7) presented in this paper.

In this paper, we focused on the landslide failure mode of TRSS which was commonly observed in the Wenchuan earthquake. Based on the results in the DEM simulation of the slopes under dynamic loading, the failure mechanism of TRSS landslide was analyzed. The stability of the Tangjiashan slope under seismic wave excitation was calculated, and the gradual failure process of the landslide was also simulated. It is found that the discontinuities distribution and strong seismic force are important reasons for the formation of TRSS. This study shows that DEM modeling is an effective way to understand the geological and mechanical mechanisms behind the landslide induced by seismic loading through reproducing the gradual disintegration process and the discontinuous characteristics of the landslide. In a word, we couple engineering geologic analysis and numerical computation to verified a seismic-induced landslide type-TRSS.

## Data Availability

Some data used during the study were provided by a third party (Sichuan Earthquake Administration (http://www.scdzj.gov.cn/)). Direct requests for these materials may be made to the provider as indicated in the Acknowledgements. The following data can be provided: Acceleration-time curves of the Wenchuan seismic wave (Horizontal: West–East component; Horizontal: North–South component; Vertical: UD component).
